# Route of administration for illicit prescription opioids: a comparison of rural and urban drug users

**DOI:** 10.1186/1477-7517-7-24

**Published:** 2010-10-15

**Authors:** April M Young, Jennifer R Havens, Carl G Leukefeld

**Affiliations:** 1 Center on Drug and Alcohol Research, Department of Behavioral Science, University of Kentucky College of Medicine, Lexington, KY, USA; 2 Department of Behavioral Sciences and Health Education, Emory University Rollins School of Public Health, Atlanta, GA, USA

## Abstract

**Background:**

Nonmedical prescription opioid use has emerged as a major public health concern in recent years, particularly in rural Appalachia. Little is known about the routes of administration (ROA) involved in nonmedical prescription opioid use among rural and urban drug users. The purpose of this study was to describe rural-urban differences in ROA for nonmedical prescription opioid use.

**Methods:**

A purposive sample of 212 prescription drug users was recruited from a rural Appalachian county (n = 101) and a major metropolitan area (n = 111) in Kentucky. Consenting participants were given an interviewer-administered questionnaire examining sociodemographics, psychiatric disorders, and self-reported nonmedical use and ROA (swallowing, snorting, injecting) for the following prescription drugs: buprenorphine, fentanyl, hydrocodone, hydromorphone, methadone, morphine, OxyContin^® ^and other oxycodone.

**Results:**

Among urban participants, swallowing was the most common ROA, contrasting sharply with substance-specific variation in ROA among rural participants. Among rural participants, snorting was the most frequent ROA for hydrocodone, methadone, OxyContin^®^, and oxycodone, while injection was most common for hydromorphone and morphine. In age-, gender-, and race-adjusted analyses, rural participants had significantly higher odds of snorting hydrocodone, OxyContin^®^, and oxycodone than urban participants. Urban participants had significantly higher odds of swallowing hydrocodone and oxycodone than did rural participants. Notably, among rural participants, 67% of hydromorphone users and 63% of morphine users had injected the drugs.

**Conclusions:**

Alternative ROA are common among rural drug users. This finding has implications for rural substance abuse treatment and harm reduction, in which interventions should incorporate methods to prevent and reduce route-specific health complications of drug use.

## Background

There has been a meteoric rise in the rates of illicit prescription opioid use and dependence in the US in recent years [1,2]. According to the National Survey on Drug Use and Health, prescription opioid nonmedical use has quadrupled in the last 20 years [3] and, among new initiates to illicit drug use, has surpassed marijuana use [4]. Further, it appears that nonmedical prescription opioid use is particularly problematic in rural areas encompassing Appalachian Kentucky, Virginia and West Virginia [5,6]. The health consequences of nonmedical prescription opioid use can be severe; long-term use can lead to physical dependence and addiction, and, at high-doses, the drugs can cause severe respiratory distress and death [7]. The motives for nonmedical use of prescription drugs are various, but studies have identified one of the most common to be individuals' desire to relieve physical pain [8]. Some evidence suggests that chronic nonmalignant pain may be greater in rural areas of the US [9], but without further research, proposed links between the rural burden of nonmalignant pain and nonmedical prescription opioid use are largely speculative. The growing burden of nonmedical prescription drug use in America and its unique manifestations in rural areas has warranted more research. For example, differences between characteristics of rural and urban prescription opioid use have been examined using data from signal detection systems [10], methadone maintenance treatment enrollees [11], probationers [12], and drug-related medical examiner cases [13]. However, to our knowledge, there are no reports on rural-urban differences in ways in which individuals are administering prescription opioids.

Route of drug administration has important implications on users' health outcomes, including risk of dependence, susceptibility to infection, and experience of route-specific health complications [14]. Injection drug users, in particular, are at a heightened risk for HIV and hepatitis C infection [15-18], drug dependence [19-21], and overdose [22]. Individual-level risk factors related to transitioning to injection drug use (IDU) from other routes of administration include unemployment [23], insecure income source [24], homelessness [23,25-27], school dropout [24], and early-onset substance abuse [28]. The extent of individuals' previous substance use [23,25] and frequency of substance use [26,27] have also been identified as correlates. A number of social and ecological factors also play a role in drug users' risk for transitioning to injection. Perceived social support or tolerance for injection [23,26], social pressure [29], and geographic proximity to dealers [30] and other IDUs [31], as well as having a friend [25], sex partner [23,32], or family member who engages in IDU [24], are also associated with transitioning to injection. Drug markets [33], drug availability [30,34], and social norms surrounding typical routes of administration, collectively referred to as "site ecology" can also play a role [27]. Temporal trends in transitions to injection sometimes precipitated by changes in drug availability have also been identified [35,36]. Non-injection routes of administration are typically more expensive in terms of 'bang per buck', thus transitioning to IDU can also entail economic motivation [35]. Previous studies have shown that drug price [30] and cost-effectiveness [27,29] can play a role in determining patterns in routes of administration as well.

Studies suggest that nonmedical prescription opioid use can involve various routes of administration, the choice of which can be influenced by demographic factors such as gender and age [37-41]. However, the influence of rurality on routes of administration for nonmedical prescription opioid use has not been explored. The purpose of this study was to describe rural-urban differences in routes of administration for: buprenorphine, fentanyl, hydrocodone, hydromorphone, methadone, morphine, OxyContin^®^, and oxycodone.

## Methods

A total of 212 participants entered the study in two Kentucky counties, one a non-metropolitan Appalachian county and the other in a metropolitan area of the state's Bluegrass region [42]. The rural county has been designated by the Appalachian Regional Commission as economically depressed [43]. Both counties are predominantly white (97.3% and 77.4%, respectively) [44].

Participants were recruited using snowball sampling, which is most commonly used to access hidden populations such as drug users [45]. In the current study, participants who were initially recruited with flyers or by community key informants who agreed to participate in the study were asked to refer additional participants, who in turn were asked to refer additional participants and so on. Participants were eligible if they reported having used any prescription opioid nonmedically in the prior 30 days and OxyContin^® ^at least once in the prior three years (either medically or non-medically). The purposive sampling of OxyContin^® ^users is a product of the purpose of the overall goal of the study, which was to compare outcomes of OxyContin^® ^use among rural and urban drug users.

Data were collected between October 2008 and August 2009. Interviewers were three research assistants who resided in the target communities. After determining eligibility and obtaining informed consent, an interviewer-administered questionnaire was utilized to gather information on socio-demographic, medical, family/social characteristics, and self-reported behaviors. The MINI International Neuropsychiatric Interview, version 5.0 [46] was used to measure the following psychiatric disorders: major depressive disorder (MDD), generalized anxiety disorder (GAD), post-traumatic stress disorder (PTSD) and antisocial personality disorder (ASPD). Drug problem severity was examined using a composite score from the Addiction Severity Index (ASI) [47]. For the purposes of the current study, participants were also asked to indicate lifetime and recent (past 30 day) use of the following substances for the purposes of getting high: buprenorphine (e.g., Subutex^®^, Suboxone^®^), fentanyl patch, hydrocodone (e.g., Norco^®^, Vicodin^®^, Lorcet^®^, Lortab^®^), hydromorphone (Dilaudid^®^), methadone tablets, morphine (e.g., MSContin^®^, Kadian^®^, Avinza^®^), OxyContin^® ^(tablets and generic), and other oxycodone (e.g., Tylox^®^, Percocet^®^, Percodan^®^). For each specific drug for which participants reported lifetime use, they were asked about the frequency of using the following routes of administration: swallowing (including swallowing whole and chewing to swallow), snorting, and injecting. Participants were interviewed in locations such as a library or other public places and were compensated $50 for their time. The study was approved by the University of Kentucky Institutional Review Board.

## Analysis

The dependent variable of interest was substance-specific route of administration (i.e. for each substance, there were three dichotomous outcomes defined by lifetime engagement in swallowing, injecting, and/or snorting as a route of administration). Categorical and continuous demographic characteristics of rural and urban drug users were compared using chi-square tests and Mann-Whitney U-tests, respectively. Logistic regression analysis was used to examine differences between rural and urban participants' route of administration, adjusting for age, gender, and race. The statistical software SPSS Version 17.0 (SPSS Inc., Chicago, IL) was used to conduct data analysis.

## Results

### Description of the sample

Descriptive characteristics of the sample (n = 212) are displayed in Table 1. Rural drug users comprised 47.6% (n = 101) of the sample. The median age of all participants was 37 years and ranged from 20 to 69. The majority of participants were men (54%) and 51% were non-Hispanic white. The median number of years of formal education completed was 12. Just under half (49%) had been employed in the past 30 days and 20% were receiving pension for disability. The median monthly legal income was $665 and most participants (59%) did not have health insurance. Just over 21% were married or remarried, 34% were widowed, separated, or divorced, and 45% had never been married. Rural participants were significantly younger, had fewer years of formal education, earned less income than urban participants, and had significantly higher drug problem severity scores on the Addiction Severity Index. Significantly more rural participants were non-Hispanic white, non-religious, and married or remarried than were urban participants.

**Table 1:** Comparison of demographic characteristics for rural (n = 101) and urban (n = 111) drug users

Descriptive characteristics	Rural ***n (%)***	Urban ***n (%)***	Total ***n (%)***	*P *value
Male	57 (58.2)	56 (50.9)	113 (54.3)	0.294
White	96 (95.0)	11 (9.9)	107 (50.5)	**< 0.001**
Age - median (IQR)	33 (27 - 43)	42 (30 - 49)	37 (29 - 47)	**0.004**
Years in county - median (IQR)	31.0 (25 - 37)	30.5 (16.5 - 43)	31.0 (23 - 41)	0.467
Years of formal education - median (IQR)	12.0 (9 - 12)	12 (12 - 14)	12.0 (10 - 12.5)	**< 0.001**
Recent legal income*- median (IQR)	$600 (300 - 800)	$720.50 (468 - 1289)	$665 (400 - 1020)	**0.003**
Employed in Past 30 Days	43 (42.6)	61 (55.0)	104 (49.1)	0.072
Receives Pension for Disability	21 (20.8)	21 (18.9)	42 (19.8)	0.733
Married/Remarried	29 (28.7)	16 (14.4)	45 (21.2)	**0.011**
Non-religious	64 (63.4)	30 (27.0)	94 (44.3)	**< 0.001**
Uninsured	57 (56.4)	68 (61.3)	125 (59.0)	0.488
Has Chronic Medical Problem	57 (56.4)	49 (44.1)	106 (50.0)	0.074
Prescribed Medication for Physical Problem	36 (35.6)	58 (52.3)	94 (44.3)	**0.015**
Ever Treated for Drug/Alcohol Problem	49 (48.5)	48 (43.2)	97 (45.8)	0.442
ASI Composite Drug Use Score - median (IQR)	0.26 (0.14 - 0.34)	0.08 (0.03 - 0.17)	0.16 (0.06 - 0.28)	**< 0.001**
Psychiatric characteristics				
Major Depressive Disorder	47 (46.5)	28 (25.2)	75 (35.4)	**0.001**
Generalized Anxiety Disorder	41 (40.6)	38 (34.2)	79 (37.3)	0.339
Post-traumatic Stress Disorder	20 (19.8)	13 (11.7)	33 (15.6)	0.105
Anti-social Personality Disorder	32 (31.7)	31 (27.9)	63 (29.7)	0.550

IQR - Interquartile range, ASI - Addiction Severity Index [47]

* Income in past 30 days from employment, unemployment compensation, welfare, pension, benefits, social security, mate, family, friends, or child support

Approximately half (46%) of participants had ever enrolled in drug or alcohol treatment. Fifty percent of the sample reported that they had a chronic medical problem and 44% were regularly taking prescribed medication for a physical problem. Significantly more urban participants were regularly taking prescribed medication for a physical problem than rural participants. Approximately 35% of participants met the DSM-IV criteria for major depressive disorder (MDD), 37% for generalized anxiety disorder (GAD), 16% for post-traumatic stress disorder (PTSD), and 30% for anti-social personality disorder (ASPD). Significantly more rural participants met criteria for MDD than did urban participants (Table 1).

### Drug Use and Route of Administration

Table 2 describes rural and urban nonmedical drug use and the routes of drug administration for each of the drugs. No urban participants reported lifetime use of buprenorphine or of the fentanyl patch. Among rural participants, however, 51% reported buprenorphine use and 37% reported fentanyl use, both of which were most commonly administered by swallowing. Interestingly, 15% of rural participants reported injecting fentanyl patch contents. Preferred route of administration varied by substance and by rural/urban status. Among urban participants, swallowing was the most common route of administration across all substances. In age-, race-, and gender-adjusted analyses, urban participants had significantly higher odds of reporting swallowing hydrocodone and oxycodone than did rural participants. Among rural participants, the preferred route of administration varied according to substance. For hydrocodone, methadone, OxyContin^®^, and oxycodone, snorting was the most frequent route of administration. Significantly more rural participants reported snorting hydrocodone, OxyContin^®^, and oxycodone than did urban participants, after adjustment for age, race, and gender. For hydromorphone and morphine use among rural drug users, injection was most common. Notably, among rural participants, 67% of hydromorphone users and 63% of morphine users had administered the drugs by injection.

**Table 2:** Age-, gender-, and race-adjusted comparisons for route of drug administration among rural (n = 101) and urban (n = 111) drug users

	Rural	Urban	Adjusted *
	%	%	***P*****-values**
Buprenorphine (sublingual tablets)	50.5	0	---
Swallowing	31.7	0	---
Snorting	26.7	0	---
Injecting	3.0	0	---

Fentanyl (patch)	35.6	0	---
Swallowing	25.7	0	---
Snorting	1.0	0	---
Injecting	14.9	0	---

Hydrocodone (tablets)	90.1	91.9	0.408
Swallowing	68.3	91.9	**0.046**
Snorting	74.3	6.3	**< 0.001**
Injecting	0	0	---

Hydromorphone (all formulations)	32.7	4.6	**0.001**
Swallowing	6.9	4.5	0.524
Snorting	5.9	0.9	0.472
Injecting	21.8	0	---

Methadone (tablets)	77.2	3.6	**< 0.001**
Swallowing	27.7	3.6	0.083
Snorting	64.4	0	---
Injecting	1.0	0	---

Morphine (all formulations)	53.5	4.6	**0.007**
Swallowing	14.9	3.6	0.652
Snorting	17.8	0.9	0.547
Injecting	33.7	0	---

OxyContin^® ^(generic/tablets)	86.1	23.6	**0.002**
Swallowing	25.7	22.5	0.442
Snorting	68.3	3.6	**< 0.001**
Injecting	44.6	0	---

Other Oxycodone** (tablets)	83.2	50.0	0.374
Swallowing	31.7	47.7	**0.026**
Snorting	68.3	1.8	**< 0.001**
Injecting	3.0	0	---

* p-values adjusting for age, race, and gender

** Includes, for example, Tylox^®^, Percocet^®^, and Percodan^®^

## Discussion

Preferred route of administration varied by substance and by rural/urban status. Among urban participants, oral use (swallowing whole or chewing and swallowing) was the most common route of administration. This contrasted sharply with substance-specific variation in routes of administration among rural participants. For example, snorting was the most frequent route of administration for hydrocodone, methadone, OxyContin^®^, and oxycodone, while injecting was most commonly used for hydromorphone and morphine administration. After adjustment for age, race, and gender, rural users had significantly higher odds of snorting hydrocodone, OxyContin^®^, and oxycodone compared to urban participants.

The increased odds of rural participants to use alternative routes of administration warrant consideration. Previous research has demonstrated that multiple routes of administration are involved in nonmedical prescription opioid use [40,41,48]. In fact, our finding on the frequency of snorting OxyContin^® ^compared to swallowing and injecting is consistent with the findings of another Kentucky study [39]. That study, conducted in a clinic-based sample from central Kentucky, found that methadone, morphine, and hydromorphone were being administered through various alternative routes, including snorting, chewing, and injecting [39].

Previous literature has posited that the decreased availability of heroin in rural areas may contribute to rural-urban differences in prescription opioid use [11-13]; however, this trend is not apparent in this sample, as nearly twice as many rural participants reported lifetime use of heroin than did urban participants (data not shown). Rather, differences in the prevalence of alternative routes of administration is likely to be more intimately linked to differences in drug problem severity. Previous substance use [23,25] and frequency of current substance use [26,27] are known risk factors for transitioning to injection from other routes of administration. Scores from the Addiction Severity Index [47] indicate that rural participants had much higher drug problem severity than did urban participants, which may have contributed to the rural/urban differences in route of administration evident in this study.

The routes of administration for buprenorphine use among rural participants in this study are consistent with other studies [37,49-52]. For example, the relative frequency of buprenorphine snorting compared to injecting in this study is interesting with implications for preventing diversion. Strategies intended to prevent buprenorphine intravenous misuse, like Suboxone^®^, may not prevent misuse by alternative routes of administration. The opiate antagonist naloxone contained within Suboxone^® ^"guards" against misuse by causing withdrawal symptoms in those who inject or snort it; however, the data are conflicting [53].

The routes of fentanyl administration by rural study participants are also noteworthy. Over 70% of rural fentanyl users administered the drug orally. Oral administration of fentanyl has been identified within other populations [38,54-56]; however, these studies have generally found oral administration to be rare in comparison with other routes of administration. Oral fentanyl administration can result in a wide range of concentrations in the blood, depending on whether the substance is retained in the oral cavity or swallowed [56,57]. Nevertheless, oral fentanyl administration can have fatal consequences, as demonstrated by findings from post-mortem studies of fentanyl-related deaths [55,56]. Injecting fentanyl, found among 42% of the fentanyl users in this study, has also been reported in other populations [55,58,59]. The frequency of fentanyl injection in this study is concerning given its implications for toxicity and overdose. A fentanyl dose that is survivable following transdermal administration may result in death if administered intravenously [55]. Deaths due to fentanyl overdose following injection can occur at low blood concentrations (2.0 μg/L - 3.0 μg/L) [55,59-61]. These results are especially disconcerting given that ambulance response times are significantly slower in rural areas [62], which may increase the likelihood of fatal overdose.

Perhaps most concerning about the high prevalence of alternate routes of administration is the potential for transmission of blood-borne infections such as HIV and hepatitis B and C. While HIV and hepatitis C (HCV) in particular are transmissible by injecting [63-65], it has also been demonstrated that HCV can be transmitted by sharing equipment used to snort drugs, such as straws [65-67]. A seminal review by Strang and colleagues (1998) discusses various health implications for route of drug use, including nasal ulceration from snorting and respiratory and thrombotic complications, abscesses, and endocarditis from injecting [14]. The health consequences of nonmedical prescription opioid use, as delivered by any route of administration can be severe, entailing potential for physical dependence and addiction, severe respiratory distress, and fatal overdose [7]. Overdose risk, in particular, is compounded by the route of administration [68]. Reports have noted that this is especially problematic in OxyContin^® ^use, which was designed to be a slow-release formulation [69].

While this study broadens understanding of rural substance abuse and alternate routes of administration for prescription opioids, it is not without limitations. The data in this study are self-reported and are subject to response bias. This study is also limited by sample size, which prohibited making statistically meaningful rural-urban comparisons for buprenorphine and fentanyl, as well as statistically precise point estimates for certain routes of administration of other substances. The rural-urban comparisons were also complicated by the baseline demographic differences between the two groups. Race-, gender-, and age-adjusted analyses were used in an attempt to isolate the influence of rurality on the outcome of interest; however, a number of unmeasured social, economic, and structural factors may have also influenced the comparison. Also, given the influence of ecological factors such as drug availability and drug price on determining routes of administration [30], the study would have been strengthened by an examination of these characteristics in the rural and urban settings involved.

## Conclusions

This study offers valuable insight into the intricacies of nonmedical rural opioid use in particular. These findings suggest that alternative routes of administration are common among rural drug users, a phenomenon which is likely related to drug problem severity. This finding has implications for rural substance abuse treatment as well as prevention of transition from oral to other routes of use such as snorting and/or injection. The presence of alternative routes of administration among rural drug users also indicates a need for the implementation of harm reduction interventions within this population.

## Competing interests

This study is funded by Purdue Pharma L.P. Points-of-view and opinions expressed in this article do not necessarily represent those of Purdue Pharma but represent the opinions of the authors.

## Authors' contributions

AY performed the statistical analysis and drafted the manuscript. All authors read and approved the final manuscript.
